# Nlrx1 regulates neuronal cell death

**DOI:** 10.1186/s13041-014-0090-x

**Published:** 2014-12-24

**Authors:** Emilie Imbeault, Tara M Mahvelati, Ralf Braun, Pavel Gris, Denis Gris

**Affiliations:** Program of Immunology, Department of Paediatrics, CR-CHUS, Faculty of Medicine and Health Sciences, University of Sherbrooke, Sherbrooke, QC Canada; Institut fuer Zellbiologie, Universitaet Bayreuth, Bayreuth, Germany; Montreal Neurological Institute, McGill University, Montréal, QC Canada

**Keywords:** Nlrx1, Cells death, Necrosis, Apoptosis

## Abstract

**Background:**

Regulation of cell death during neurodegeneration is one of the key factors that play a role in the speed at which a disease progresses. Out of several cellular pathways responsible for this progression, necrosis and apoptosis are situated on the opposite spectrum of cell death regulation. Necrosis produces an environment that promotes inflammation and cytotoxicity and apoptosis is a highly organized process that maintains tissue homeostasis. A recently discovered protein, Nlrx1, regulates inflammatory and cell death responses during infection.

**Findings:**

Using transfections of N2A cell line, we demonstrate that Nlrx1 redirects cells away from necrosis and towards an apoptotic pathway following rotenone treatments. In addition, Nlrx1 promotes DRP1 phosphorylation and increases mitochondrial fission.

**Conclusion:**

Our results suggest a novel molecular pathway for regulating mitochondrial dynamics and neuronal death. Nlrx1 may play an important role in neurodegenerative diseases, where necrosis is a prominent factor.

## Introduction

Neuronal cell death is a fundamental process that governs development and homeostasis of the central nervous system (CNS) [1]. During development many neurons die off in the process of pruning, which leaves only those neurons that have meaningful connections. Throughout adult life, neurons have to survive under constant environmental stress such as toxins, infections, and inflammatory mediators. Inability to cope with these stimuli results in neuronal cell death and neurodegeneration that lead to neurological dysfunction [2]. There are three major types of cell death: necrosis, apoptosis, and autophagy. Necrotic cell death is the least controlled process that triggers cellular pathways, which leads to bursting of cells and leakage of the internal materials (such as HMGB1) in the extracellular environment. This leakage is highly cytotoxic and induces robust pro-inflammatory responses. Apoptosis is an organized step-like process that initiates with nuclear condensation, membrane blebbing, and leads to formation of apoptotic bodies that are phagocytized by microglia and astrocytes. Finally, autophagy may be considered a cell survival pathway as it mobilizes cell resources in response to many stress events including inflammation, starvation, hypoxia, etc. Driven to extreme, autophagy may lead to cell death [3]. Remarkably, mitochondria is situated at the crossroads of all three pathways, and thus regulate the balance between the three types of cell death [4]. Mitochondria are well known for their ability to induce apoptosis by releasing cytochrome c and by activating downstream caspases. In addition, mitochondrial fusion and fission are critical to the survival of neurons. Interestingly, mitochondrial fission was shown to be protective during ischemia and during Huntington’s disease [5].

Inflammation is an integral part of the tissue response to any kind of cell death. This response may become cytotoxic and even damaging to surrounding cells depending on the milieu. For example, during infection or tissue damage, microglia and astrocytes are activated by pathogen-associated molecular patterns (PAMPs) and danger associated molecular patterns (DAMPs). Once activated, these cells release cytokines and chemokines that attract more inflammatory cells. In addition, they release reactive oxygen and nitrogen species thus, increasing the cytotoxicity of the environment and leading to excessive neuronal cell death. The concentrations and compositions of PAMPs and DAMPs are monitored by sensors and receptors including Toll-like receptors (TLRs), NOD-like receptors (NLRs), RIG-I helicases (RLRs), etc. [6,7]. Multiple proteins from the NLR family regulate intestinal homeostasis, regulating susceptibility to inflammatory bowel diseases and cancer [8].

Of these receptors, Nlrx1, belongs to the NLR family of intracellular sensors that regulate major cellular pathways including cell death and inflammation. Previous research implicated Nlrx1 in the regulation of autophagy and reactive oxygen species production during viral infection [9]. In addition, most recent publications implicated Nlrx1 in the regulation of cell death, gastritis, and colon cancer [10–13]. Moreover, we demonstrate that Nlrx1 modulates neuronal apoptosis by regulating mitochondrial fission.

## Materials and methods

### Chemical reagents

BRD, Mdivi, staurosporine, and rotenone were purchased from Sigma-Aldrich. Z-VAD FMK was purchased from R&D systems. MitoTracker Mitochondrion-Selective Probes were purchased from Invitrogen. Trizol was purchased from Life Technologies. M-MLV Reverse Transcriptase and RNasin Ribonuclease Inhibitor were purchased from Promega. Oligo(dT) primer was purchased from Fermentas Life Sciences and PCR Nucleotide Mix was purchased from GE Healthcare. Brilliant III Ultra-Fast SYBR Green QPCR Master Mix was purchased from Agilent Technologies. α/β-Tubulin rabbit, cleaved caspase-3 rabbit, DRP1 rabbit, phospho-DRP1 (Ser616) rabbit, HSP90 Rabbit, HMGB1 Rabbit, COX IV Rabbit, and anti-rabbit IgG HRP-linked antibodies were purchased from Cell Signaling Technology. Nlrx1 polyclonal antibody was purchased from Proteintech.

### Cell culture and cell lines

All cell lines were generated on the basis of mouse neuroblastoma (N2A) cells. Nlrx1 stable Knock-In N2A cells were generated using Origene TrueORF cDNA Clone Nlrx1 vector system with TurboFectin. Nlrx1 stable Knock-Down cells were generated using Origene ShRNA plasmid against Nlrx1. The vector contains a tGFP gene, which expresses tGFP constitutively in mammalian cells. Transfection stable control was generated using a GFP Scrambled ShRNA from Origene. Nlrx1 Knock-In GFP positive cells were selected with neomycin and Nlrx1 GFP positive Knock-Down and Scrambled were selected with puromycin. Real time Quantitative PCR and RT-PCR was used to verify expression of Nlrx1. Primers sequences *Nlrx1* F: 5′-CCT CTG CTC TTC AAC TTG CTC-3′, *Nlrx1* R: 5′-CCC ATC TGA TCC AGA ACA TCG-3′, 18S F: 5′-CGG CTA CCA CAT CCA AGG AA-3′, 18S R: 5′-GCT GGA ATT ACC GCG GCT-3′ were purchased from IDT.

### Experimental procedures

#### Western blotting

Membranes were incubated with primary antibody (1:1000) overnight at 4°C and secondary (1:2000) for 2 hours at room temperature.

#### Cell death assay

Cell death was detected by LDH release with a microtiter plate based colorimetric absorbance assay that was developed based on a protocol from Chan and al, 2013.

#### Flow cytometry

Mitochondrial mass was evaluated using Mitotracker Mitochondrion-Selective Probes. 2×10^5^ cells were resuspended in 200 μl of media containing 100 nm of Mitotracker and were incubated at 37°C for 20 minutes. Samples were analyzed by flow cytometry using a FACS Calibur. Data were analyzed using FlowJo software.

#### Electron microscopy

Cells were fixed using standard protocol by glutaraldehyde in sodium cacodylate followed by osmic acid and Epon 3 impregnation. Images were collected using Hitachi H-7500.

#### Statistical analyses

Mean values were compared using Two-way ANOVA followed by Tukes’ test for comparison; significance was accepted at p < 0.05.

## Results

**Figure 1:**
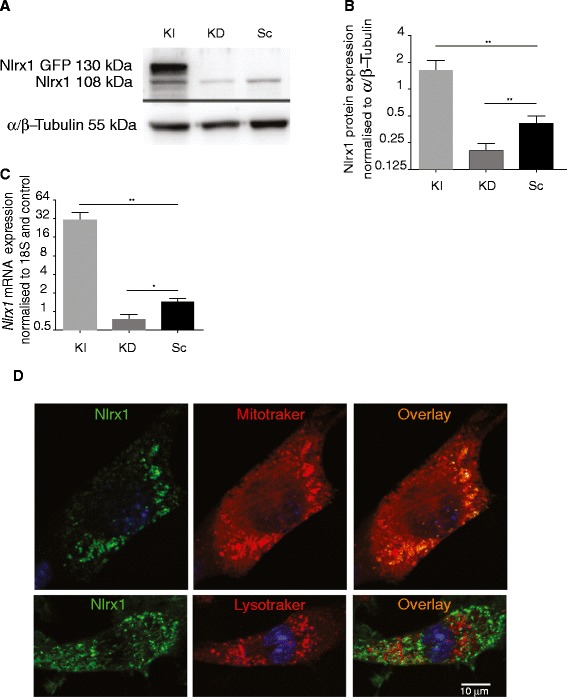
**Nlrx1 expression in N2A cells. (A)** Representative photograph of Immunoblotting of KI, KD, and Sc cells, molecular weight of endogenous Nlrx1 is 108 kDa. In KI cells Nlrx1-GFP is around 130 kDa. **(B)** Nlrx1 protein expression in KI, KD, and Sc cells, *n = 3.* **(C)** *Nlrx1* mRNA expression in KI (Nlrx1GFP), KD (sh*Nlrx1*), and Sc (Sh Scramble), *n = 5.* **(D)** Localization of Nlrx1 protein to mitochondria showing that Nlrx1 co-localized with mitochondria, but not with lysosomes. Photomicrographs of confocal images of cells labeled green for Nlrx1 and stained with mitotracker or lysotracker (red). Overlay showing co-localisation of Nlrx1 and mitotracker or with lysotracker (orange). * p < 0.0001.

To investigate the role of Nlrx1 in neuronal death, we generated Knock-In (KI) and Knock-Down (KD) N2A stable cell lines, which expressed high or low amounts of Nlrx1 respectively. Cells transfected with scrambled ShRNA (Sc) served as controls. First, we validated the expression pattern of Nlrx1 in different cell lines. We observed significant increase of expression of Nlrx1 protein and *Nlrx1* mRNA levels in KI cells compared to cells transfected with empty vector (Figure 1A, B, and C). In cells that were transfected with *Nlrx1* ShRNA, we saw two-fold reduction of Nlrx1 protein and mRNA expression. Nlrx1 was localized to mitochondria, but not to lysosomes (Figure 1D).

**Figure 2:**
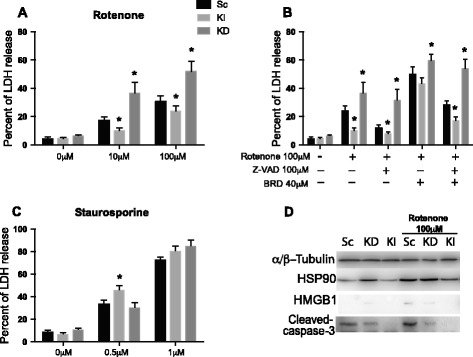
**Nlrx1 protects from rotenone-induced cell death. (A)** Cells were treated with 10 μM and 100 μM rotenone for 24 hours, *n = 3.* **(B)** Cells were treated for 24 h with 100 μM rotenone 100 μM Z-VAD FMK, and 40 μM BRD, *n = 3.* **(C)** Staurosporine treatment for 24 h kills all cells equally with exception of Nlrx1 KI cells at 0.5 μM concentration, *n = 3.* **(D)** Western blotting for markers of necrosis HSP90 and HMGB1 and marker of apoptosis active cleaved-caspase-3. * p < 0.001.

Cells were then treated with rotenone; a compound acting on mitochondrial respiration (it blocks complex I of the mitochondrial respiratory chain) and also it is implicated in the etiology of Parkinson’s disease. The release of lactate dehydrogenase was quantified, which upon cell death leaks out of the cells and into the supernatant/cell culture medium. We observed a significant rotenone dose-dependent increase in cell death in all cell lines. In addition, we noted a dose-dependent protection effect of Nlrx1, where Nlrx1 KI cells were the least affected followed by cells with WT levels of Nlrx1 in Sc cell line. The KD cell line, with decreased levels of Nlrx1, was the most vulnerable to rotenone treatments (Figure 2A). The addition of BRD (ROS enhancer [14]) to the rotenone treatment resulted in increased levels of released LDH. The relationship between the cell lines remained similar to rotenone treatment. When we used pan-caspase inhibitor Z-VAD, we observed a significant reduction of cell death in all cell lines except for Nlrx1 KD (Figure 2B). We used staurosporine as one of the widely used reagents that induces cell death via intrinsic apoptotic pathway. We noticed a significant induction of LDH release in all cell lines (Figure 2C). We did not observe significant differences in LDH release between different cell lines at 1 μM concentration of staurosporine. At 0.5 μM staurosporine, KI cells released significantly more LDH.

**Figure 3:**
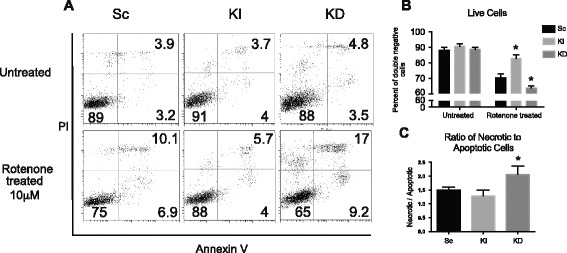
**After rotenone treatment, overexpression of Nlrx1 increases cell viability and shifts cell death to apoptosis rather than necrosis.** Flow cytometric analysis of annexin V-PI staining of cells treated with 10 μM rotenone for 24 hours. **(A)** Representative flow cytometry plots of annexin V/PI stained cells. Annexin V negative PI negative quadrant defines live cells, annexin V positive PI negative defines cells that are at the early apoptotic stages, and annexin positive I positive defines cells that undergo necrosis or in the late apoptotic stages. **(B)** Quantifications of three independent experiments. **(C)** Graphical representation of ratios of necrotic/late apoptotic cells to early apoptotic cells, *n = 3*. * Significantly different from Sc p < 0.01.

The release of LDH is an indirect indicator of cell mortality; therefore, we used Western blotting for active cleaved-caspases-3 in cell lysates as an indicator of the degree of apoptosis. HSP90 and HMGB1 presence in cell culture medium were used as indicators of necrosis. The basal levels of HSP90 and HMGB1 were significantly higher in KD cell line after 24 hours of 100 μM rotenone treatment. We observed significantly smaller amounts of activated caspase-3 and HMGB1 as well as HSP90 in KI cells compared to Sc and KD cell lines (Figure 2D). Furthermore, to evaluate cell viability and the degree of apoptosis, we used flow cytometry of annexin V/PI stained cells (Figure 3A). We observed a significant decrease in live cells after rotenone treatment (Figure 3B). KI cells were significantly more resistant to the rotenone treatment. Thus, to understand the balance between necrosis and apoptosis in cells following rotenone treatment, we evaluated the ratio of cells in the upper right quadrant, which defines cells in the stage of late apoptosis or necrosis, to the cells in lower right quadrant, which defines cells in early stages of apoptosis. We found that the ratio in KD compared to KI cells was significantly higher (Figure 3C).

**Figure 4:**
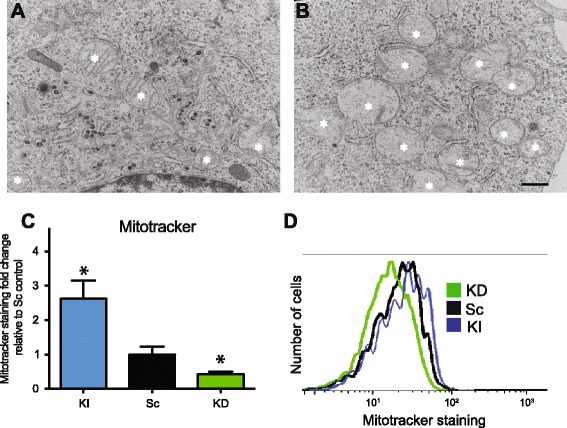
**Nlrx1 promotes mitochondrial fission.** Photomicrographs showing mitochondrial content. **(A)** KD Nlrx1 cells have a decreased mitochondrial number. **(B)** KI Nlrx1 cells have more mitochondria and those mitochondria have swollen cristae. * represent mitochondria, scale bar is 500 nm. **(C)** There is a higher mitochondrial mass in KI Nlrx1 cells compared to KD Nlrx1 cells as showed by Mitotracker red staining in GFP positive cells, *n = 3.* **(D)** Representative plot of flow cytometry experiment with Mitotracker red staining showing a higher mitochondrial mass in KI Nlrx1 cells compared to KD Nlrx1 cells.

**Figure 5:**
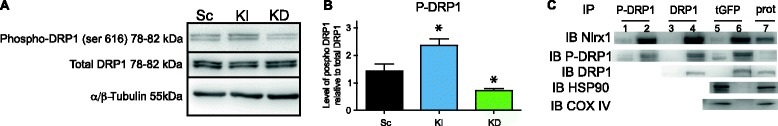
**Nlrx1 associates with DRP1. (A)** Immunoblotting of DRP1 and Phosphorylated DRP1 at serine 616 in Sc, KI, and KD N2A cells. **(B)** Quantification of phospho-DRP1 normalised to total DRP1. There is more phosphorylated DRP1 at Ser616 in KI Nlrx1 cells compared to KD shRNA and Sc cells. **(C)** Immunoprecipitation of Nlrx1 and DRP1, but not of Nlrx1 and HSP90 or COX IV. Co-immunoprecipitation with anti-tGFP antibody in lane 5 and 6; and with anti-DRP1 antibody lane 3 and 4; and anti-P-DRP1 lane 1 and 2. Lane 1, 3, and 5 contain supernatant; lane 2, 4 and 6 contain IP fractions, and lane 7 contains total protein lysate. The Western blot was probed with anti-DRP1 antibody. * Significantly different from Sc, p < 0.05.

Hence, we hypothesised that mitochondrial localisation may enable Nlrx1 to regulate mitochondrial dynamics. Using electron microscopy (Figure 4A and B) and flow cytometry (Figure 4C and D), we observed an increased number of mitochondria in KI cells compared to KD as well as to control cells. Additionally, KI cells’ mitochondria were swollen and had less cristae (Figure 4A and B). The process of increasing mitochondrial number is called fission and is governed by multiple proteins including DRP1. DRP1, in particular, mediates fission upon phosphorylation of Ser616. We verified the expression and phosphorylation status of DRP1 in different cell lines and observed significant increases in phosphorylation of DRP1 in KI compared to KD and to control cells (Figure 5A and B). Then, we performed immunoprecipitation using antibodies against DRP1 and P-DRP1 in order to pull DRP1; and we used anti-GFP to pull Nlrx1. We then probed blots with anti-DRP1, anti-P-DRP1, anti-Nlrx1, anti-COX IV (mitochondrial protein), and anti-HSP90 antibodies. We observed an association whenever we used anti-DRP1, anti-P-DRP1, and Nlrx1 antibodies, but not when anti-COX IV and HSP90 were used (Figure 5C).

**Figure 6:**
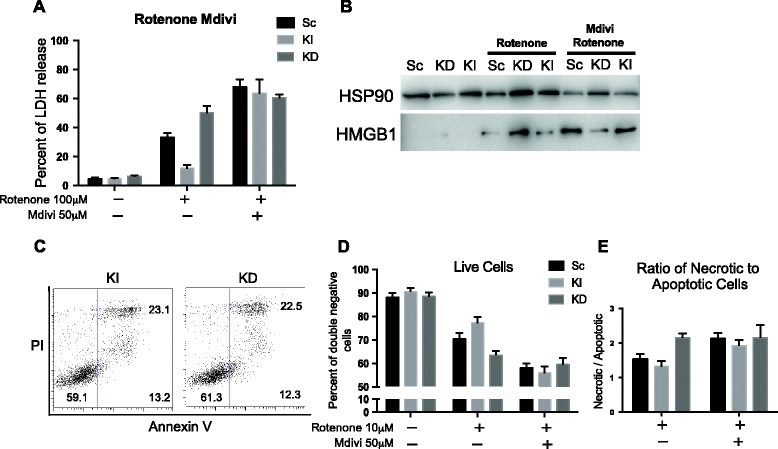
**DRP1 inhibitor Mdivi contracts Nlrx1 protective effect after rotenone treatments. (A)** Quantification of released LDH assay after treatments with 100 μM rotenone and 50 μM Mdivi for 24 hours, *n = 3.* **(B)** Representative Immunoblotting of HMGB1 and HSP90 in cell culture supernatants after rotenone and Mdivi treatments. **(C)** Representative flow cytometry plots of KI and KD cells treated with 10 μM rotenone and 50 μM Mdivi. **(D)** Quantifications of three independent experiments. **(E)** Graphical representation of ratios of necrotic/late apoptotic cells to early apoptotic cells. * Significantly different from Sc, p < 0.05.

These results, so far, suggest that Nlrx1 may regulate cell survival by associating with DRP1 and thus augmenting mitochondrial fission. To verify DRP1-dependent effect of Nlrx1, we treated all cell lines with Mdivi, a DRP1 inhibitor, in addition to rotenone treatment. Using LDH assay, we observed that Mdivi abolished protective effect in KI cells, but had little effect in KD cells (Figure 6A). These results were confirmed by Western blotting, where we observed a significant induction of necrosis in KI cells after combined Mdivi and rotenone treatment (Figure 6B). Furthermore, Mdivi treatment equalized the amount of live cells and ratios of necrotic to apoptotic cells across all cell lines (Figure 6C, D, and E).

## Discussion

The field of NLR biology is young and the majority of research has been directed towards the role of NLRs in the host-pathogen interaction. Nlrx1 is one of the few NLRs that, in addition to mediating the immune response, regulates cell death in multiple cell types. In this report, we provide evidence that Nlrx1 controls cell death by regulating the mitochondrial homeostasis. In particular, we found that Nlrx1 augments mitochondrial fission that protects cells from the rotenone toxicity. We found that Nlrx1 protects N2A cells during necrosis-like cell death but not against reagents like staurosporine that potentially induce apoptotic cell death. While in absence of Nlrx1, cells are more protected against apoptosis-inducing stimuli, they are more sensitive to necrosis. In deciphering Nlrx1’s molecular pathway, we found that Nlrx1 associates with DRP1, which augments mitochondrial fission and thus saves cells from necrosis. Indeed, the inhibition of DRP1 resulted in loss of Nlrx1-mediated protection during rotenone-induced cell death.

Overexpression of Nlrx1 in N2A cells significantly reduced rotenone-mediated cell death, while reduction of Nlrx1 made cells more vulnerable to rotenone toxicity. Previous research suggested that Nlrx1 may mediate ROS production [15,16]. We used BRD treatment that enhances non-toxic ROS production. Although this treatment increased rotenone-dependent cell death, the effect in the different cell lines was similar, which suggests that mechanisms of Nlrx1 neuroprotection are not ROS dependent. Rotenone can induce mitochondrial dysfunction, increase in ROS production, and an increase in caspases-dependent apoptosis. At the same time, cytotoxic events within cells initiate necrosis [17–19]. Our results suggest that Nlrx1 inhibits both rotenone-induced necrosis and apoptosis. Indeed, after rotenone treatment, we observed reduced presence of HSP90 and HMGB1 in the supernatants from KI cells compared to KD cells. In KD cells, low levels of Nlrx1 allowed cell to shift towards necrosis, which was most notable when apoptosis was inhibited by Z-VAD.

Our observations are also confirmed by another study from Girardin group who found that Nlrx1 accelerates intrinsic apoptotic pathway [10]. In that paper, Nlrx1 augmented intrinsic apoptotic pathway while inhibiting TNF cyclohexamide-sensitive cell death. In a different report, authors demonstrated that TNF may induce necrotic programed cell death mediated through TNFRI RIP2 TRAF2 [20]. Allen et al. demonstrated that viral infection induces Nlrx1-mediated autophagy in cells. Interestingly, another recent report found that Nlrx1 protects macrophages by blocking the function of the viral proteins that induce apoptosis. Another group demonstrated that Nlrx1 is mediating virally-induced autophagy, but they did not report an effect of Nlrx1 on cell death [21]. Our work suggests that in the absence of viral infection, Nlrx1 redirects cellular stress towards apoptosis thus, protecting cells from necrosis-like cell death. We did not notice any physiological or biochemical differences between Nlrx1 KI and Nlrx1 KD cells at basal level suggesting that Nlrx1 functions are triggered only during stressful conditions. These results are collaborated by multiple studies with Nlrx1 KO mice. Although, Nlrx1 has been implicated in many cellular pathways, Nlrx1 KO mice are viable and fertile and do not show any deviations from WT mice at the basal conditions [16,22–24].

Several groups have shown Nlrx1 to localize to mitochondria, although the exact distribution of Nlrx1 within the inner and outer mitochondrial membrane is still under debate.

Electron microscopy studies enforced by flow cytometry experiments suggest an increased number of mitochondria in Nlrx1 KI cells. We demonstrated that increase in Nlrx1 expression resulted in augmented mitochondrial fission with an upsurge in phosphorylated levels of DRP1. Several reports suggest that Nlrx1 may bind and regulate functions of mitochondria-localized proteins including MAVS and UQCRC2 [25,26]. The exact molecular pathway that phosphorylates DRP1 is still under investigation. Overexpressing Nlrx1 resulted in the increased number of mitochondria, but these mitochondria had a reduced number of cristae of which all were swollen, which suggests that excessive fission induced mitochondrial stress. In our opinion, those mitochondria are more sensitive to cytotoxic events, which explains why Nlrx1 KI cells were more sensitive to some of the apoptosis inducing reagents. This observation is collaborated by several studies, which evaluate mitochondrial function in cell death. A dysfunction of DRP1 and altered mitochondrial fission led to a switch from apoptotic to necrotic cell death [27]. In addition, an increase in mitochondrial fission has been implicated in the etiology of neuronal cell death in Huntington’s disease [5].

In conclusion, to our knowledge this work describes for the first time the involvement of Nlrx1 in mitochondrial dynamics during neuronal death. We would like to note that these experiments were conducted in N2A transformed cells lines and that these cells possess neuronal-like properties. Future studies will confirm this observation in primary neuronal cultures and in transgenic mice.

## Abbreviations

CNS: Central nervous system
Sc: Scrambled Sh RNA transfected cells
KD: Nlrx1 Sh RNA transfected cells
KI: Nlrx1-GFP transfected cells
NLR: NOD-like receptors
ROS: Reactive oxygen species
